# The Evidence of Different Learning Environment Learning Effects on Vocabulary Size and Reading Comprehension

**DOI:** 10.3389/fpsyg.2018.01914

**Published:** 2018-10-30

**Authors:** Yang Dong, Jieyi Hu, Xiaoying Wu, Haoyuan Zheng, Xu Peng

**Affiliations:** ^1^Department of Social and Behavioral Sciences, City University of Hong Kong, Kowloon, Hong Kong; ^2^Department of Curriculum and Instruction, The Education University of Hong Kong, Tai Po, Hong Kong; ^3^Guangzhou No.11 People’s Hospital, Mental Health Service Center, Guangzhou, China; ^4^Department of Education, College of Education Science, Jiaying University, Meizhou, China

**Keywords:** reading comprehension, receptive vocabulary, learning environment, immersion, depth of processing

## Abstract

The nature of the effect of learning environments’ language setting on second language receptive vocabulary acquisition in both adolescent receptive vocabulary acquisition and reading comprehension performance was explored in a continuous 10-month longitudinal study. The current study divided 170 adolescents into four groups. Their reading comprehension ability and receptive vocabulary size were each measured in two different periods. The results showed that single Chinese instructional learning and single English instructional learning contributed more to students’ receptive vocabulary size than bilingual instructional language materials. The results imply that the immersion hypothesis has more positive impact on improving second language receptive vocabulary size acquisition and reading comprehension performance than the depth of processing hypothesis.

## Introduction

Vocabulary size is a fundamental factor of understanding text (Lü et al., 2015). While existing research has investigated the overall advantage of immersion education in terms of learning languages, few research studies of immersion education focused on second language (L2) vocabulary development, especially in two totally orthographically different languages (e.g., Chinese and English). Immersion education refers to “immersing” students in an intensive L2 instructional environment, such as bilingual teachers using L2 as a medium of instruction. However, as depth of processing hypothesis points out, learning without additional elaboration may not properly enable students to retain their expanding vocabulary for extended periods of time. In the context of L2 learning, there are currently no studies that compare the two learning conditions mentioned below: immersing students in the written form of learning and the elaboration of learning. Therefore, little is known about the effectiveness of either of the methods in vocabulary development, especially for students with poor linguistic knowledge and low socioeconomic standings.

The aims of the present study are as follows. Firstly, we will attempt to examine the effectiveness of immersion education in vocabulary development. Contrary to previous studies of putting students in L2 instructional environments, the experimental group of students in the present study will only be immersed with L2 explanation to learn target vocabulary for homework assignments. Secondly, we will also compare the effectiveness of immersion education and elaboration of vocabulary. Finally, vocabulary gained in the three settings will be investigated in relation to its association with reading comprehension.

## Literature Review

### The Foundation of Vocabulary on Reading Comprehension

Vocabulary is recognized as a significant component of reading comprehension in second language acquisition (SLA). Theoretically, the lexical restructuring model suggested by Walley et al. (2003) indicated an indirect positive relationship between vocabulary and reading development. Consequently, it is this restructuring that facilitates reading development. Vocabulary has fundamental influence on students’ overall language proficiency (Yuksel and Kavanoz, 2010). Recently, many empirical studies have proven that L2 vocabulary has a positive association with students’ understanding of written text and even has one of the strongest positive correlations with learning L2 reading comprehension (see meta-analysis in Jeon and Yamashita, 2014). This positive association was found among learners at different educational levels (Schmitt et al., 2011; Li et al., 2012; Zhang, 2012; Quinn et al., 2015). Over time, vocabulary was a key predictor for reading comprehension for early L2 learners (Lervåg and Aukrust, 2010). In addition, vocabulary enhancement was also beneficial for minority students in reading comprehension (Lesaux et al., 2010) in terms of understanding different texts (Rydland et al., 2012).

As shown in the mental lexicon model proposed by Levelt et al. (1999), individuals can store vocabulary through the lexical representation of the phonology or sound patterns (lexical form) and word meaning of the lexicon (meaning level). Therefore, vocabulary knowledge can be differentiated into two categories: breadth and depth of vocabulary. Breadth of vocabulary refers to “the number of words whose meaning has at least some superficial knowledge” (Qian, 2002). Many studies have shown a positive relationship between receptive vocabulary knowledge and reading comprehension (Qian, 1999, 2002; Farvardin and Koosha, 2011; Moinzadeh and Moslehpour, 2012; Yunus et al., 2016). Moreover, a large size of receptive vocabulary may help learners to retain more words in the long-term (Webb, 2007).

### Environment

#### The Effect of Environment on Vocabulary

Environmental factors have significant impact on learning second languages, especially learning vocabulary (Collentine, 2004) and reading comprehension (De Jong and Leseman, 2001). The environment impacts vocabulary learning through the degree of authenticity of the L2 environment (Collentine, 2004; Dewey, 2004; Hakansson and Norrby, 2010) and the home literacy environment (Reese et al., 2000; Suter, 2006). For the degree of L2 exposure, the influence on students’ vocabulary development is unclear. Evidence among studies has failed to prove the existence of significant advantages in studying abroad over studying domestically in terms of the development of vocabulary knowledge (Dewey, 2004), proper written vocabulary usage (Freed et al., 2003), and speed of vocabulary acquisition (Collentine, 2004).

A student’s home environment may also account for the student’s vocabulary development. A majority of studies showed that predictors such as a family’s socioeconomic status (SES; Reese et al., 2000) and the literacy activities parents engaged in with their children (Hart and Risley, 1995) may be related to literacy development.

#### The Effect of Environment on Reading Comprehension

Environments also influence learners’ reading comprehension in both first language (L1) and L2 (De Jong and Leseman, 2001). For L2 learning, external factors such as home literacy environment and instructional environment may have influence on students’ reading development (Dreyer and Nel, 2003; Reese et al., 2008; Netten et al., 2016; Yao and Renaud, 2016). Instructional environments may also have impacts on learners’ reading abilities such as reading time and accuracy (Yao and Renaud, 2016) and use of reading strategies (Dreyer and Nel, 2003).

Many studies examined environments by investigating their effects on either vocabulary or reading comprehension. Few research studies attempted to examine environmental effects on both variables. Moreover, fewer studies tried to compare the L2 authentic environment with a mixed language learning environment in terms of their effects on vocabulary development and reading comprehension. The present study tried to examine whether immersing students in a high level of L2 authentic environment would help them in acquiring vocabulary knowledge and promoting reading comprehension. This study would also measure learning gains when presenting students with bilingual form-meaning. These two types of environmental settings would be compared and analyzed based on their effectiveness of vocabulary learning and reading achievement.

### Immersion

Immersion education refers to immersing students in an intensive L2 instructional environment (Swain and Johnson, 1997) by using L2 as a medium of instruction. Students are taught many or all of the academic subjects in L2. In this case, L2 is learned with no assistance in learners’ native language. Students are expected to learn L2 in this immersive situation, incidentally, where the focus is academic content instead of learning the language itself (Genesee, 1985). With regard to within-group designs, the immersion program is shown to have a positive impact on students’ vocabulary acquisition (Thériault, 2015). In terms of between-group designs, many studies have shown that immersion programs benefit L2 vocabulary development more than regular L2 learning (Barik and Swain, 1976; Genesee, 1978; Harley and Jean, 1999; Lo and Murphy, 2010).

Despite the evidence showing the beneficial impact of immersion programs on vocabulary development, it should be noted that many of these programs were tailored specifically toward Europe and the United States, and the first languages of these students were generally European languages. When a study traced back to the origin of these language, it was found that many of the English or German words were generated on the basis of Latin, from which French was also developed (Andersson and Janson, 1991). Therefore, students might find it easier to learn orthographically similar words. However, for languages that show more significant difference in orthography, such as Chinese and English, it would be much more difficult in comparison for students to shorten their proficiency gap while being immersed in an intensive L2 environment.

Furthermore, there is no study, so far, that examines the association of vocabulary gained in immersion program and reading comprehension. The majority of the existing studies mainly focus on investigating the vocabulary size.

In addition, according to the features of immersion education, second languages (e.g., English) are used as the language of instruction in the classroom. According to the lexical quality hypothesis, skilled readers have demands of high-quality lexical representations (Perfetti and Hart, 2002). In other words, robust word knowledge helps learners to recognize the printed words. It is implied that reading comprehension requires higher quality lexical representations in comparison with the comprehension of speech. When students are immersed in oral interaction, the lexical items acquired from communication might not be enough for the comprehension of print.

Moreover, previous studies on immersion education involved teachers tailoring the instruction in classrooms. Teachers, who deliver the input, might be an additional factor affecting students’ vocabulary learning in terms of quality input (White, 1987). For example, teachers might not be aware of their students’ current abilities. Thus, input delivered by teachers might be inappropriately difficult for students to understand the meanings of vocabulary (e.g., Tian and Hennebry, 2016). Also, there might be a tendency for teachers with lower English proficiency to be unclear about the specified lexical explanation. With teachers’ involvement, it might be unclear whether the specific learning environment might truly affect students’ cognition when learning vocabulary. Therefore, this study was carried out by immersing students in L2 explanation through homework assignments. This could exclude the potential effect of teachers’ influence on the environment.

### Mental Effort Required for Vocabulary Learning

Some researchers argue that additional elaboration is necessary for learners to store the vocabulary in their long-term memory. Cognitive psychologists propose that the storage of the information depends on how deeply this information is processed in our brain (Craik and Lockhart, 1972; Ellis, 2010). The depth of processing hypothesis indicates that information stored in memory depends on how shallow or deep the information processing is initially. Similarly, the retention of vocabulary may be greater when learners learn it through deeper elaboration or richness of semantic processing (Laufer and Hulstijn, 2001).

In SLA research, scholars have tried various methods to lead learners’ attention/mental effort to the target words with elaboration by the use of glosses and dictionaries. Many empirical studies succeeded in eliciting the effectiveness of texts with gloss compared with no-gloss for students’ vocabulary enhancement (Hulstijn et al., 1996; Watanabe, 1997). However, when studies examine the differences of L1 and L2 glosses, it generates conflicting results. Laufer and Shmueli (1997) discovered that learners with the use of L1 glosses outperformed those with L2 glosses. Nevertheless, Miyasako (2002) found that L2 glosses benefited students more in terms of vocabulary learning than L1 glosses. Moreover, in Xu’s (2010) examination on L1, L2, and L1 plus L2 glosses, it was shown that L1 glosses tended to be more beneficial for vocabulary learning than both L2 glosses and the combination of both. Many empirical studies showed the effectiveness of the use of dictionaries during reading toward enhancing vocabulary learning (Hayati and Fattahzadeh, 2006; Ahangari and Dogolsara, 2015). However, there is no consensus as to the types of dictionary use for vocabulary development. In Laufer and Hill (2000) and Laufer and Hadar’s (1997) study among high school and university students, learners, regardless of their education level, scored higher in vocabulary tests with the use of bilingual dictionaries rather than bilingual and monolingual dictionaries.

### The Current Study

As can be seen in previous studies, additional efforts or elaboration proved to be beneficial for learners in learning and retaining the target lexical items and in understanding of texts. However, there remains a question about the types of elaboration that would have positive impact on students’ learning. Vocabulary in the previous studies has been regarded as an assistant for learners to understand the text or paraphrase rather than the prime task of learning the target vocabulary without the accompaniment of the text. With the text provided, it may not be clear whether reading materials may help learners to infer the meaning of the words and, thereby, improve their understanding of the text. A hypothesis in the current study is that different learning environmental settings may contribute different learning results to reading comprehension and vocabulary size.

Hypothesis 1: L2 words with L2 explanations contribute to a better learning result for vocabulary size and reading comprehension than the results obtained by using L2 words with L1 explanations.

Hypothesis 2: L2 words with both L1 and L2 explanations result in the best learning outcome for vocabulary size and reading comprehension.

## Materials and Methods

### Participants

This study selected 170 Chinese students in grade seven from four “Hongzhi” classes in the south east part of China. The “Hongzhi” class represents the students who have significant academic ambitions and who recognize education to be a prerequisite to future success in life. All students were from mountainous rural areas and had excellent scores in the entrance exam across all subjects other than English. Students scored poorly in the English section of the entrance exam. All of them were in mountain-village Hukou, and the school offered them studentship for both tuition fees and normal daily expenditures.

All participants regarded Mandarin as their L1 and English as their L2. There are 42 students in Class One (21 boys and 21 girls, Mean_age_ = 11.57 years, *SD*_age_ = 0.50 years), 44 students in Class Two (16 boys and 28 girls, Mean_age_ = 11.55 years, *SD*_age_ = 0.50 years), 42 students in Class Three (20 boys and 22 girls, Mean_age_ = 11.55 years, *SD*_age_ = 0.50 years), and 42 students in Class Four (17 boys and 25 girls, Mean_age_ = 11.45 years, *SD*_age_ = 0.50 years). All participants had similar Raven scores (*p*s > 0.98) and entrance exam scores of other subjects also were similar (*p*s_chinese_ > 0.87, *p*s_math_ > 0.98). All participants had already mastered around 685 words, which equates to a 0–2-year-old child’s English level in native English-speaking countries, and they were able to understand basic sentences in Longman Contemporary English Dictionary (Horst and Collins, 2006).

Class One to Class Three were taught by one researcher and Class Four was taught by a trained teacher. These two teachers had 3 years of English teaching experience in middle school. All English teaching programs were the same in that the curriculum outline and teaching content were offered by the team of English teachers within the school.

### Measures

Participants were required to take four tests in total. Two of the tests were for testing vocabulary size and the other two were for testing reading comprehension.

#### Reading Comprehension

Two reading comprehension scores were selected from the English entrance exam of first year students and final English test of grade seven. For the reading comprehension portion, the total score was 40. The difficulty indicator was 0.5, which means about 50% of the students could get scores of at least 24 for this section. The test was provided by the City Education Bureau and was also used for all students in the representative city. All difficulty indicators for the final English exam in this academic year were the same for normal students. The internal reliability for each comprehension test was over 0.850.

#### Receptive Vocabulary Size

The Receptive Vocabulary Size Test was selected with a scale from the study conducted by Liao (2012), in which the version was revised from Juel’s (1988) work. Liao’s scale consisted of three levels (each level had 10 words to represent that level’s vocabulary size) of word vocabulary size for middle school students in Mainland China. The first part represented at most a frequency of 1,000 words while the second part tested the 2,000 words level, and the third part represented the 3,000 words level (500 words were the requirement of English teaching syllabus, and another 500 were words beyond the syllabus). The frequency of using words decreased gradually from part one to part three. Only when the participant answered at least nine questions correctly within a 1000 words level, could he move to the next level portion of the exam. If a participant chose a correct answer for a question, they received one point, otherwise they received zero points. Students were required to attend part one first.

Here is an example of the words level test:

“See”: They saw it. A. cut; B. waited for; 3. looked at; 4. started. The internal reliability was 0.92.

### Procedures

The current study followed a quasi-experimental design. All vocabulary lists were selected from the requirements of the China New English Curriculum (2015). Researchers copied and pasted the words’ explanations from the Longman dictionary in three forms, Form One as English words with English explanation (EE) (sentence) only, Form Two as English words with EE (sentence) and Chinese explanation (CE) (short terms), and Form Three as English words with CE (short terms). Class 1, 2, and 3 took Form One, Two, and Three, respectively. For example, when the target word is “appreciate,” it was represented in the following ways: Form One: “appreciate: used to thank someone in a polite way or to say that you are grateful for something they have done”; Form Two: “appreciate: used to thank someone in a polite way or to say that you are grateful for something they have done, 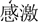
”; and Form Three: “appreciate: 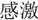.”

### Research Procedures

At the beginning of semester A, this study requested consent forms from students and their parents in the first academic week. In the second week, the research carried out a series of tests and collected student performance scores from test papers and their entrance exam papers. All participants were required to attend the vocabulary test. Starting in week two, we began by dividing four classes into four subgroups. Group One to Group Three were experimental groups, using English words with EE, English words with both English and Chinese explanation (ECE), and English words with CE, respectively. Group Four was the control group without any intervention.

Next, during school days, the teacher assigned 10 new words from the syllabus as the day’s homework for students. There were 3,000 words in total. The exceptions to this were the 55 holidays from the beginning of semester A (time one) to the middle exam in semester B (time two). Students were required to write down the given words every day and submit them to their English teachers on the next school day. Time two data collection was carried out about 10 months later, which was around the time of the final exam for grade seven. All participants were required to test their vocabulary sizes again during this time. Accordingly, the researcher later took students’ reading comprehension scores from the normal English exam.

### Data Analysis

We used SPSS 22.0 to perform data analysis. Firstly, we provided the descriptive analysis, including the mean vocabulary size for each group, the standard deviation of vocabulary size for each group, and the maximum score and minimum score for each group. Secondly, in order to examine the effect of the three experimental learning environmental settings, we set four groups as dummy variables, and we regarded the control group as the controlled variable. After setting the dummy variables for all three learning environments, we provided the zero-order person correlation matrix for the examination of the correlation among the three learning environments, vocabulary size, and reading comprehension. Later, we further analyzed the contributing effect of the three learning environments on reading comprehension and vocabulary size; we conducted linear regression analysis between the three learning environmental settings and reading comprehension and vocabulary size.

## Results

The results are presented in two main sections. First, we provide the descriptive statistics and the interrelations between our variables (learning environment, vocabulary size, reading comprehension). Next, we present the regression analyses relating to the two research hypotheses. The first set of aims confirm whether or not the L2 learning environment receives better results for vocabulary size and reading comprehension than the native language learning environment. The second aim is to determine whether the immersion learning theory is stronger in L2 vocabulary size enlargement and reading comprehension improvement than the mental effort theory.

All participants completed every task at each time point. The data presented here includes two-time wave collection. During Time One, which was at the beginning of the class arrangement test, no significant differences in English vocabulary size scores and English reading comprehension ability scores were found between the students of the four classes continuing at Time Two.

### Descriptive Statistics and Interrelations Between Variables

The means, standard deviations, and range of scores for English vocabulary size and English reading comprehension at Time One and Time Two are shown in Table 1. All analyses were conducted using raw data. From Table 1, at Time Two, the EE group’s students received the highest score for the vocabulary size and reading comprehension tests. The EC group’s students received the second highest scores and were followed by the ECE group’s students. The control group’s students received the lowest score.

**Table 1:** Descriptive analysis.

		Mean		SD		Minimum		Maximum	
					
		Pre	Post	Pre	Post	Pre	Post	Pre	Post
EE (*n* = 42)	Vocabulary size	604.76	2411.90	88.21	136.51	500	2200	700	2600
	RC	18.31	34.69	1.22	3.17	16	30	20	40
ECE (*n* = 44)	Vocabulary size	604.55	1709.09	83.40	215.47	500	1300	700	2000
	RC	18.41	28.82	1.09	4.64	17	22	20	37
EC (*n* = 42)	Vocabulary size	614.29	1830.95	81.37	153.79	500	1600	700	2200
	RC	18.31	31.32	1.18	2.98	16	26	20	39
Control (*n* = 42)	Vocabulary size	595.24	1350.00	76.36	207.48	500	1000	700	1700
	RC	17.93	25.95	1.14	12.31	16	23	20	38


*RC = reading comprehension test.*

### The Association Between Environmental Settings and Learning Outcomes

Learning groups were represented by dummy variables while the control group was set as the control variable. The concurrent correlations are shown in Table 2. As seen here, all correlations are significant at the specified level (*p*s < 0.001). The EE group received the highest correlation indicator in both vocabulary size and reading comprehension scores, while the ECE group received the lowest correlation indicators. In each environmental learning group, the correlation indicator was higher in vocabulary size than the reading comprehension correlation.

**Table 2:** Correlation effect on learning environment, vocabulary size, and reading comprehension.

	1	EE	ECE	EC
(1) Vocabulary size		0.95^∗∗∗^	0.65^∗∗∗^	0.80^∗∗∗^
(2) Reading comprehension	0.62^∗∗∗^	0.85^∗∗∗^	0.37^∗∗∗^	0.70^∗∗∗^


*All beta indicators are in significant level, *p* < 0.001.*

### The Coefficient of Determination of Environmental Settings in Learning Outcomes

There were two central aims of this set of analyses. The first purpose was to compare the learning result in a single language learning context. The second aim was to test whether or not more information offered in the learning environment contributed to better results. First, we created four dummy environment variables for each environmental setting. We, then, performed linear regression to measure the power of each environmental setting.

Table 3 shows that the three learning environmental settings positively predicted vocabulary learning outcome and reading comprehension learning outcome. The EE group had the most power while the ECE group had the least power in determining vocabulary size enlargement and reading comprehension ability. The three learning environmental settings would explain the vocabulary learning outcomes as 90, 64, and 42%, respectively. The three learning environmental settings determined the reading comprehension learning outcomes as 72, 48, and 12%, respectively.

**Table 3:** Effect of three learning environments on reading comprehension and vocabulary size.

	Vocabulary size			Reading comprehension		
		
	B	SE	R^2^	B	SE	R^2^
EE	1061.91	38.23	0.90	8.74	.60	0.72
EC	480.95	39.85	0.64	5.17	0.58	0.48
ECE	359.09	45.65	0.42	2.87	0.80	0.12


*All beta indicators are significant at the 0.001 level, *p* < 0.001.*

## Discussion

### The Benefits of Foreign Language Environments Over Native Language Translations

Exposing students to the L2 environment could benefit them more in receptive vocabulary development and reading comprehension than the EC condition. Moreover, the result of significant gains in reading comprehension in immersion condition was in line with previous findings (Gebauer et al., 2013). This indicates that immersion contributed to academic reading in L2-English. The immersion context might provide three conditions that promote the development of vocabulary and reading comprehension. These include the inhibition of L1, the enhancement of L2, and new ways of thinking. The participants in our EE group were immersed in L2 when they were doing homework. This process took place for nearly 1 year. During this time, learners’ L1 was inhibited while they were limited to EEs with L1 translations. Learners in the EE group were forced to learn the target vocabulary with the EE. Students under elaborated exposure (form-meaning exposure with many variables) tended to have better word retention (Eckerth and Tavakoli, 2012). Moreover, previous studies showed that the length of exposure to new cultural settings along with new language learning might change individuals’ way of thinking. This could include divergent (Kharkhurin, 2008), analytic, and holistic styles of thinking (Bell, 1995). It might be possible that students in immersion settings underwent a process that influenced a different way of thinking among students, and this was reflected in their higher scores for reading comprehension compared with the students in other groups.

### The Benefits of a Single Learning Environment and a Mixed Language Environment

Our result also showed that the English-English environment had the highest correlation indicator in vocabulary size and reading comprehension, whereas the English-Chinese-English group had the lowest correlation indicator. This implies that the single learning environmental setting enhances SLA more than the mixed language condition. These existing results could be interpreted from three perspectives.

Learning in a pure L2 environment might be more effective than that in a mixed language environment. The highest correlation observed in the EE environment might be explainable by the Revised Hierarchical Model (RHM; Kroll and Stewart, 1994). According to RHM, when learners are at the beginning of learning L2, they are more likely to connect L2 words with the concept through the mediation-L1 representation. With more proficiency in L2, a more direct route of L2 and concept would be linked as learners no longer need to rely on their L1 representation. As a result, the link to L1 becomes weaker in comprehension (e.g., reading and listening) and production (e.g., writing and speaking). According to the language-specific selection model (Costa et al., 1999), there is an activation system in individuals’ cognition. When applying this to our study, the signals or cue provided in the present study were the EEs. The frequency of reading and using L2 might be higher. The cues for L1, including the present materials and their knowledge about L2, could not be matched with L1 because of their poor proficiency. They were not strong enough for the EE group of learners to activate L1. Consequently, the link between L2 and L1 lemma representation would not be strong enough to establish the necessary memory connection.

Another hypothesis was based on the effect of L1 inhibition on weakening the link between L2 and L1 representations. The lexical-conceptual links under such resistance of L1 might be facilitated and strengthened at the expense of reducing dependency on L2-L1 lexical links. When students were required to repeatedly write and recite the English vocabulary in English, their L1 lexical representation might not have a proper place to function. Such an interpretation was incompatible with the shift of bilingual lexicon in a mixed learning environment. When students were exposed to an L2 explanation along with an L1 translation, they might tend to tie the concept of L2 to the L1 lemma because of their low L2 proficiency (Costa and Santesteban, 2004). When L1 was provided, learners might focus on the L1 short-form of explanation more frequently than the L2 explanation. Therefore, the indirect route from L2 to the concept was still strongly mediated by the L1 representation, and this led to a slower progression of learning.

Immersion in a pure L2 environment might trigger individuals’ depth of processing within their cognition. Although significant gains were observed in the immersion program, it might not be appropriate to simply deny the effectiveness of depth of processing. In this case, we argue that there might be a possibility that a certain level of depth of processing was activated within the immersion environment. Consequently, this process might strengthen the vocabulary knowledge gains through the facilitation of retention. Previous studies that focused on either L1 or L2 learning showed that information that was more difficult to comprehend or took a greater amount of effort tended to be more memorable (McDaniel et al., 1986; Griffin and Harley, 1996). Other researchers (Laufer and Hulstijn, 2001) used a “high involvement load” to denote such a condition that requires a greater amount of learner’s mental effort. Thus, there would be a possibility that students in the EE group also experienced multiple levels of depth of processing while immersed in the L2 environment such as difficulty levels and encoding levels (e.g., association and elaboration).

In terms of difficulty levels, when learners with a low level of L2 proficiency initially encountered new L2 words with L2 explanations, they might find it more difficult to associate the L2 terms with the existing L1 representations than students provided with L1 plus L2 explanations. As a result, learners in the EE group might need to try to decode the meaning of the L2 terms by remembering the L2 synonyms in order to develop the connection between L2 and concept.

With regard to encoding levels, when students enter the encoding phase, there might be association and elaboration processing. As for association, it was more likely that they might associate the new information with their existing knowledge by trying to find out a similarity or by constructing a contrast between the old information and new information. Moreover, once the association was established, learners might try to think about how this new information could be integrated into their L2 network. The level of elaboration might also take place. Many existing literatures revealed that the level of elaboration during processing determines the improvement in memorizing words. Learners with poor proficiency might try to understand and comprehend the sentences or phrase first, before making connections between L2 and concept. Finally, they might also attempt to construct the meaning to form relations at the end. In mixed language environments with both L1 and L2 explanations, although there were many different materials, students’ attention might not be diverted to all the entries as they have multiple options among the L1 words and L2 explanations.

### The Overall Effects of Learning Environmental Settings on Vocabulary Size and Reading Comprehension

The correlation analysis and linear regression model result showed that the environmental setting had a larger correlation with vocabulary size than with reading comprehension. This result could be interpreted from two dimensions.

#### Learning Environmental Settings Effect on Vocabulary Size and Reading Comprehension

First, as for training methods, the primary purpose of the training was to help students learn the L2 vocabulary in different environmental settings. There was no training involved in teaching students skills to improve their reading comprehension. Therefore, it was not surprising to find out that the scores of the vocabulary tests in all groups were higher than those of the reading comprehension tests.

Next, task complexity in vocabulary and reading comprehension tests were different. The vocabulary tests were designed with matching multiple choice questions for students to choose the meaning of the target vocabulary. However, text comprehension requires individuals to form a meaning-based understanding of the text/paraphrase (Kintsch, 1988). Reading comprehension is a process that involves knowledge, either from a linguistic understanding of the text or world knowledge. It could be said that the output of the vocabulary test was less complex than that of the reading comprehension test. According to the Limited Attentional Capacity model (Skehan and Foster, 1997; Robinson, 2001), individuals’ attentional resources are limited. The attentional capacity is based on working memory. Moreover, the performance of the L2 output is dependent on the complexity of the language, accuracy, and fluency. To prioritize one perspective for enhancement, other perspectives might be hindered. To conclude, learners might struggle in understanding the coherence and integration of the text, leading to no other attentional resources for producing the answer correctly and fluently. However, in vocabulary tests, learners only matched target words with correct answer in a multiple choice environment. This task was less complex and less cognitively demanding. This might result in learners paying a sufficient amount of attention to linguistic forms.

### Environmental Effects on Vocabulary

Another hypothesis was that the enhancement of reading comprehension might not be associated with environmental settings but with different vocabulary knowledge gains from the environmental settings. Previous studies showed that vocabulary plays a significant role in reading comprehension for L1 (Lervåg and Aukrust, 2010) but also for L2 (Jeon and Yamashita, 2014). Findings showed that the number of words an individual knows in the passage determined their reading comprehension scores (Schmitt et al., 2011). Moreover, growth of vocabulary seems to predict the development of reading comprehension (Quinn et al., 2015). Therefore, we could not deny the fact that environmental settings interact with vocabulary gains to affect reading comprehension. This means that the learning environment might impact vocabulary learning directly and impact reading comprehension indirectly. Students in immersion environments tended to gain more vocabulary, and this led to higher reading comprehension scores. Students in other groups, on the other hand, had lower gains of vocabulary knowledge, which hindered their reading comprehension growth. Environmental settings, however, only had effects on different vocabulary sizes.

Another hypothesis could be that the interactive effect of the environment and vocabulary was not enough to promote reading comprehension. According to the Direct and Inferential Mediation Model (DIME), although the effect of prior knowledge (e.g., home literacy environment) on reading comprehension could lead to better reading comprehension, the inference skills for students to bridge the gap from sentences to sentences and between prior knowledge and the current text was crucial for the development of reading comprehension. Previous studies showed that training in drawing inference could help learners with comprehending the text better (McKeown et al., 2009) by adding explicit referential association (Gilabert et al., 2005) and asking learners to explain their understanding (Ainsworth and Burcham, 2007). Within our study, there was no inference skill training. When conducting the reading comprehension test, learners might not be able to use extra-textual cues (de Bot et al., 1997). They might also fail to recognize significant words that demonstrate the author’s attitude, fail to have accurate preconceptions about the possible meaning of the words (Frantzen, 2003), and fail to connect their prior knowledge with the text.

## Implications

With regard to theory contribution, there were some points worthy of mention. Firstly, this study managed to show that to facilitate the L2 learners’ mental connection between the L2 words and their concept, immersing learners in a pure L2 language environment (homework situation) proved to be the most powerful condition in abridging and strengthening the link by inhibiting the route of L2 to L1 equivalent representation.

Secondly, this study was the first to compare the effectiveness of a pure L2 environment (immersion) and a mixed language environment (depth of processing) in homework situations. Our findings contributed to the existing literature that it was possible that a mechanism of depth of processing was involved in immersion conditions. In addition, multiple levels of encoding the L2 explanation were involved, such as associating the similarity and contrast of the existing knowledge and elaborating the meaning through sentences. One might argue that the L1 condition plus the L2 condition might have similar effects. Nevertheless, we should not neglect the fact that the provision of the L1 explanations could help learners to have a short-cut to memorize the vocabulary by associating the link between the L2 and L1 representations. Therefore, students might tend to use the L1 explanation to learn the specified vocabulary.

Thirdly, another important point worthy of mention was that we failed to argue that the effectiveness of depth of processing was more or less significant than the effectiveness of immersion in homework situations. This was because the design of the study might involve variables that reduce the effect of depth of processing in the L1 and L2 groups, such as learners’ shifts of the L1 lexicon to associate the L2 words. However, we attempted to argue that there might be more levels of depth of processing in immersion situations than in mixed language environments, and these processes seemed more effective for learners when learning vocabulary.

Fourthly, the interactive effect of the environmental setting on vocabulary might impact reading comprehension, but there were other additional factors such as inference skills that made key contributions. Therefore, future research should focus more on the crucial factors in promoting learners’ reading comprehension.

In terms of educational practice, there were three points of recommendation for teachers and educators to take note of. These included reducing the use of L1 and promoting an L2 environment; inducing more levels of processing in the L2 environment; and integrating inference skills in instruction. Firstly, when assigning homework for students with poor L2 proficiency, teachers could immerse them in a pure L2 language environment. Various methods could be adopted for the reduction of the use of L1. Moreover, it was highly recommended that in order to facilitate students’ L2 learning, teachers should request students to use English to English dictionaries for vocabulary look-up in order to not only promote vocabulary learning but also train their western way of thinking. Secondly, teachers could try to trigger students’ deeper level of processing. This could be achieved through input and output processing. For example, when assigning homework, teachers could also include some exercises asking students to write one sentence with the given vocabulary. Moreover, only immersing students in an L2 environment might not be sufficient for them to have better reading comprehension. Therefore, teachers might need to provide additional classes to integrate the knowledge of inference, such as classes on how to estimate the meaning of the words within the text and how to associate their prior knowledge with the text.

## Limitations and Suggestions for Future Research

It is important to point out some limitations of the present study. To begin, the first limitation could be that we could not ask students in the EE group to participate in the EC or ECE groups. It was obvious that the participation of the same group of students in three conditions could reduce variables. Future research could try to use this method to replicate the findings in a more accurate way.

The second limitation might be that we failed to observe or survey students on whether or not they were exposed to other learning materials, especially for the group of immersion students. There was the possibility that students in the immersion group could have utilized bilingual dictionaries to learn the vocabulary when they encountered difficulty. Future research could use observation methods or administer questionnaires to elicit the effects of other variables.

The third limitation was that depth of processing might exist among students in the immersion group. However, we failed to examine whether or not this mechanism existed under the immersion condition. Moreover, we did not know what types or degrees of the depth of processing were involved. In the future, researchers could use focus-group interviews to elicit the in-depth cognition involved in the target students (Thomas et al., 1995, as cited in Rabiee, 2004). Immersion could facilitate the connection between the L2 words and the concept. However, this study lasted for 10 months. It is unknown when and if the threshold of this connection diverges. In addition, we still did not know whether or not this inhibition would still exist or if it would be eliminated because of the stronger link of L2 to conception. According to previous neural science studies, Perani et al. (2003) showed that the effect of different exposures could impact early bilinguals. Future research should be tailored to examine whether this impact would take place in domestic immersion.

Additionally, in this study we failed to examine the correlation between vocabulary size and reading comprehension. There is a possibility that the interactive effect of environmental settings and vocabulary size is a significant predictor of reading comprehension. Future studies could attempt to examine the correlations of vocabulary and reading comprehension and compare the effectiveness of environmental conditions and vocabulary size on reading comprehension.

The fifth limitation was that all participants came from the Guangdong province in China, which might not represent other provinces in China. Participants’ SES, educational background, and other factors might be different in different provinces.

Finally, the tools we used to measure vocabulary size for pretest and posttest were a limitation. We chose multiple choice questions for students to select the meaning of vocabulary. It is possible that students might guess the meaning and randomly select the answer even though they did not truly know the meaning of the words. Therefore, researchers, in the future, could try to use other forms of vocabulary tests, such as form-meaning link for learners to produce the target forms for given meanings (Schmitt et al., 2001).

## Conclusion

Immersion (requiring students to learn vocabulary through L2 explanation) could be more effective than the mixed language environment in terms of vocabulary learning and reading comprehension. Moreover, it is argued that a certain degree of depth of processing took place when students learned vocabulary in the immersion group. The level of the depth of processing in the immersion group might be greater than that of the mixed language group. In addition, environmental settings might have a greater impact on vocabulary development than on reading comprehension. Future studies could focus on examining whether or not the environment had an effect on reading comprehension.

## Ethics Statement

This study was carried out in accordance with the recommendations of the Ethical Guidelines Jiaying University, The Ethical Committee of Jiaying University. The protocol was approved by the (name of committee). All subjects gave written informed consent in accordance with the Declaration of Helsinki.

## Author Contributions

YD provided the most crucial idea for this study and wrote most parts of this paper. JH contributed a lot in terms of data analysis support, the most important part in an empirical study. XW, HZ, and XP were responsible for collecting the data, searching the materials, and revising the paper.

## Conflict of Interest Statement

The authors declare that the research was conducted in the absence of any commercial or financial relationships that could be construed as a potential conflict of interest.
